# Are There Any Differences in Road Traffic Injury Outcomes between Older and Younger Adults? Setting the Grounds for Posttraumatic Senior Personal Injury Assessment Guidelines

**DOI:** 10.3390/jcm12062353

**Published:** 2023-03-17

**Authors:** Flávia Cunha-Diniz, Tiago Taveira-Gomes, Agostinho Santos, José Manuel Teixeira, Teresa Magalhães

**Affiliations:** 1 Faculty of Medicine, University of Porto, 4200-319 Porto, Portugal; 2 Center for Health Technology and Services Research (CINTESIS@RISE), Faculty of Medicine, University of Porto, 4200-319 Porto, Portugal; 3 Toxicology Research Unit, University Institute of Health Sciences, Advanced Polytechnic and University Cooperative (CESPU), CRL, 4585-116 Gandra, Portugal; 4 MTG Research and Development Lab, 4200-604 Porto, Portugal; 5 Faculty of Health Sciences, University Fernando Pessoa (FCS-UFP), 4249-004 Porto, Portugal; 6 National Institute of Legal Medicine and Forensic Sciences, 3000-213 Coimbra, Portugal; 7 Porto Health Care Unity—Accidents, Fidelidade—Insurance Company, 4100-207 Porto, Portugal

**Keywords:** road traffic accident, medico-legal evaluation, injury assessment, damage assessment, older adult, elderly

## Abstract

Injury outcomes seem to be more severe in older than younger persons. This may make personal injury assessment (PIA) particularly difficult, mainly because of seniors’ previous health frailties. To set the grounds for seniors’ PIA guidelines, we compared an older with a younger adult population of trauma victims and, secondarily, identified differences between the groups regarding *three-dimensional* and medico-legal damage parameters assessment. Using a retrospective study of victims of road traffic accidents, we compared the groups (n = 239 each), assuring similar acute injury severity (ISS *standardised difference* = 0.01): G1 (older adults); G2 (younger adults). Logistic regression was used to estimate the odds ratio. G1 revealed higher negative consequences when considering the *three-dimensional* damage assessment, with more frequent and severe outcomes, being a cause of further difficulties in daily living activities, with a loss of independence and autonomy. Nevertheless, regarding the medico-legal damage parameters, *permanent functional disability* did not show significant differences. This study generates evidence that reveals the need to rethink the traditional methodology of PIA in older persons, giving more relevance to the real-life contexts of each person. It is essential to: obtain complete information about previous physiologic and health states, begin the medico-legal assessment as early as possible, make regular follow-ups, and perform a multidisciplinary evaluation.

## 1. Introduction

Ageing can be defined as the standard, predictable, and irreversible changes of various body systems over time. These physiological changes generally result in the loss of functional reserve in most body systems [1,2]. Healthy ageing is the process of increasing and preserving physical and mental capacities and the functional ability that enables well-being in older age [3,4]. Not all elderly individuals are frail, and to better assess the ability to recover from trauma and injuries, an assessment of the frailty of the older victim should be carried out [5,6]. The frailty syndrome is defined to evaluate the health status of older people in whom there is a progressive age-related decrease in functional physiological reserve and a decline in various body systems, presenting a low resistance to stressors and causing vulnerability and a worse response to adverse events, which is also associated with several critical chronic diseases, such as cardiovascular and pulmonary diseases and diabetes. Among the frailty markers are decreased muscle mass, strength, endurance, balance, and walking performance, as well as low activity [3,7]. The frailty syndrome is catalogued in the International Classification of Diseases—10th revision (ICD-10) under the code R54—Senility, which includes senescence, senile asthenia and debility, excluding age-related cognitive decline (R41.81), senile psychosis (F03), and sarcopenia (M63.84) [8].

**Figure 1:**
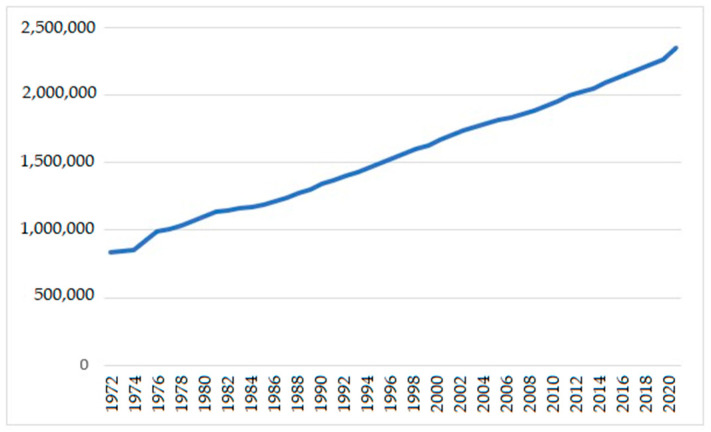
Evolution of the population aged 65 and over in Portugal [13].

Population ageing is about to become one of the most significant social world transformations, with cross-cutting implications for all sectors of life. Worldwide, the population aged 65 and over is growing faster than all younger age groups [4,9,10,11]. This older age group currently constitutes 21% of the inhabitants of the European Union and represents 23.4% of the Portuguese population (while in 1970, it was only 9.7%) [12,13] (Figure 1).

In fact, scientific and medical advances, as well as improvements in living conditions, contribute to the increase in life expectancy and allow older adults to be progressively more active and autonomous [14]. They now have more mobility, walking and travelling more frequently and for longer distances when compared to previous cohorts [15,16,17,18]. Therefore, they are more often victims of road traffic accidents (RTAs) [15,16,19].

The main reasons for increased trauma rates, namely, related to RTAs in elderly individuals, are the decline in physical capacities, with decreased sensory, psychomotor, and cognitive skills, associated with decreased reaction speed, slowed pace, poor vision and audition, reduced reflexes, and vehicle speed calculation error [1,16,20,21,22,23].

RTAs in senior victims differ in their characteristics and outcomes from RTAs in young and middle-aged adult victims [24]. The energy needed to cause injuries decreases as the person’s age increases, having more severe consequences than in younger people; road accidents occur because some particularities of this age group are related to more frequent health fragilities [25]. Older adults generally suffer less serious trauma mechanisms, although the risk of severe and critical injuries is more considerable, causing significant morbidity and mortality, which increases with age [17,18,24,26,27,28,29,30]. Furthermore, the elderly population usually presents health problems before RTAs due to the anatomical deterioration of structures, physiological frailties (with decreased physiological reserve, reduced immunity, and decreased bone and neuromuscular strength, in addition to decreased effectiveness in metabolic and endocrine responses), specific comorbidities, and even previous traumas. All this is a cause of reduced efficacy of compensatory mechanisms after an RTA trauma, impairing healing [1,14,19,31,32]. In seniors, even low-severity injuries can destabilise the previous physical and psychological states, causing important outcomes [5,14,15,20,23,32,33,34].

Owing to these aspects, trauma in senior persons is generally associated with: (a) a more extended period of hospitalisation, particularly in intensive care units [4,23,35]; (b) a more significant posthospitalisation decline in functional status, with a reduction in their quality of life, often with loss of their independence and autonomy [4,23,35]; and (c) an acceleration on senile evolution, with significant deterioration and increased functional disability after trauma, leading to a state of dependence that did not previously exist [4,33].

All these facts create great complexity and challenges in older persons’ health care and personal injury assessment (PIA), but the scientific evidence in this field is very scarce. This is stated in a critical article that resulted from the first multidisciplinary *Consensus Conference on Medico-Legal Assessment of Personal Damage in Older People*, held in 2019 [36]. This article considers the need for the development of formal guidelines on this subject and identifies four thematic areas that need further research: (a) differences in injury outcomes in older people compared to younger people and their relevance in PIA; (b) pre-existing status reconstruction and evaluation; (c) medico-legal examination procedures; and (d) multidimensional assessment and scales.

Therefore, the general objective of our study is to set the grounds for senior people’s PIA guidelines, comparing an old adult with a young and middle-aged adult population of RTA victims. The secondary objectives are to identify differences between both populations regarding: (a) *body*, *functional*, and *situational* outcomes (*three-dimensional damage* assessment); and (b) Portuguese medico-legal damage parameters assigned to the cases.

## 2. Materials and Methods

### 2.1. Data Collection Methodology

This is a retrospective study using a convenience sample based on medico-legal reports of PIA cases. The report inclusion criteria were as follows: (a) final medico-legal report about adult victims of RTA (≥18 years old), showing that the link between trauma and injuries was established; (b) performed at a health care unit of a Portuguese insurance company; (c) between 2018 and 2020; (d) by three selected physicians, with a specialisation in forensic medicine and great experience in PIA, to assure data reliability (they are all aligned with the official Portuguese rules on this subject). We did not consider the victim’s sex, the accident type, or the type of insurance responsibility—with or without fault—at this stage.

A database was created for the study and was completed by one of the physicians who performed the medico-legal assessment of the cases. No information was included that could allow those involved to be identified.

Two age groups were considered: (a) G1 (case sample)—senior adults (≥65 years old, because according to the United Nations, in developed countries, people are classified as elderly when they are 65 years of age or more [10]) (n = 239; 50%); this sample size corresponds to all available data of older adults, considering the defined inclusion criteria, namely, the time span of the study and the final PIA reports performed by the three selected physicians during the studied period; (b) G2 (control sample)—young and middle-aged adults (18–64 years old); initial cases were sorted out from all available data of this age group, considering the same inclusion criteria of G1 (n = 431); using *Propensity Score Matching* from SPSS, we found the final control sample (n = 239; 50%).

**Figure 2:**
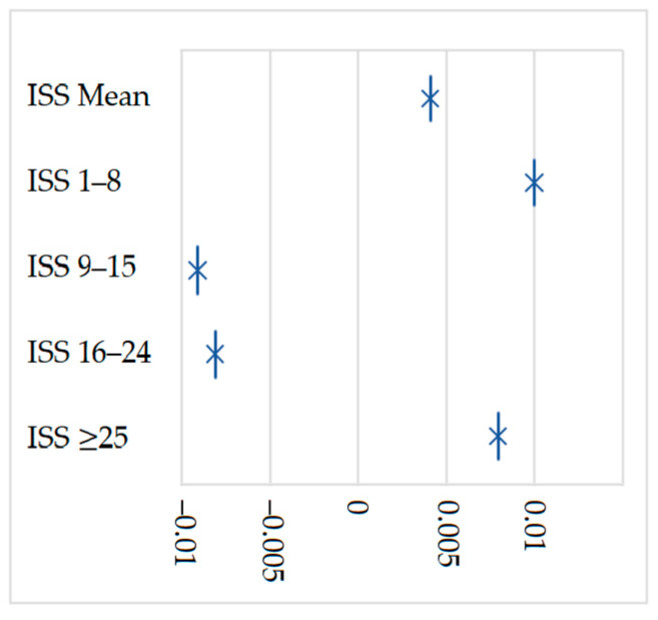
Matched predictors’ *standardised difference* (propensity score matching).

The *injury severity score* (ISS) [37,38] was used as a predictor to ensure that G1 and G2 presented a similar initial picture after the RTA. ISS was retrospectively estimated in the acute phase of the cases, considering the clinical records. The ISS variables were categorised into four classes, as shown in Table 1. To verify if the matched sample was comparable, we used the *standardised difference*, which is considered balanced when it is ≤0.1 [39]. Thus, as shown in Table 1 and Figure 2, our samples are balanced, considering the clinical severity of the cases (ISS).

### 2.2. Assessment methodology

A *three-dimensional methodology* (*body*, *functional* and *situational* levels) was used to describe permanent outcomes according to the official Portuguese rules [40,41,42].

To quantify permanent outcomes severity, we used the *Inventory for Handicap Assessment* [35], a validated instrument for Portuguese victims of RTAs, but only up to 65 years of age. However, we chose it because it is a medico-legal instrument intended for PIA purposes and because no other instrument has been validated for the older population until now. It allows us to quantify the severity degree of the *body*, *functional* and *situational* levels, and the *damage coefficient.* This *coefficient* corresponds to the average of the final scores resulting from each scale of three referred levels and considers five severity groups of increasing severity. The meaning of each level is [25,35,42]:

1. *Body* level: assesses biological outcomes, which may include morphological, anatomical, histological, physiological, and even genetic particularities;
2. *Capacities*/*functions* level: assesses physical and mental capacities (current or potential), taking into account age and sex, irrespective of the live setting;
3. *Life situations*/*participation*/*activities* level: assesses the confrontation (concrete or potential) between those affected and the reality of their physical, familial, social, cultural, educational, and professional environment.

To quantify the different parameters of damage in civil law, we used some of the Portuguese medico-legal permanent damage parameters [40,41]:

(a) *Total Temporary Functional Deficit*: period (days) in which the victim is prevented from autonomously performing acts of daily, family, and social life (without any reference to professional activity). Mostly corresponds with hospitalisation time;
(b) *Partial Temporary Functional Deficit*: period (days) in which the victim may resume activities of daily, family, and social life with some degree of autonomy, although still with limitations;
(c) *Quantum Doloris*: physical and psychic suffering experienced by the victim during the period of temporary damage on a 7-point scale of increasing severity;
(d) *Permanent Functional Deficit* (PFD): definitive effects on the victim’s physical and/or psychic integrity, with repercussions on functions and situations (daily life activities, including family and social life, leisure, and sporting activity, although it is independent of professional activities). The evaluation utilises a table of permanent disability (Decree-Law 352/2007, 23rd October, annexe 2), which uses a 100-point scale of increasing severity. PFD was categorised as 0, 1–9, 10–19, 20–39, and 40–100, drawing on the case distribution and the severity groups;
(e) *Future Damage*: damage that is not yet observable in the PIA moment but whose development is sure, corresponding to an aggravation of the sequelae in the future and consequent aggravation of specific damage parameters, namely, PFD;
(f) *Permanent Aesthetic Damage*: repercussion of the sequelae on the victim’s self-image and image in terms of others; evaluated on a 7-point scale of increasing severity;
(g) *Permanent Repercussion on Sporting and Leisure Activities*: impossibility or difficulty of the victim engaging in certain leisure, physical, or social activities that he or she regularly did and which represented a clear source of personal fulfilment and gratification; evaluated on a 7-point scale of increasing severity;
(h) *Permanent Needs*: these correspond to the victim’s needs, with repercussions on his or her independence and autonomy; they should be assessed considering the victim’s best chances of rehabilitation and reintegration.

Regarding *Temporary Professional Repercussion* and *Permanent Professional Repercussion*, we did not analyse them because, in G1, only 16 persons were professionally active before the RTA. We also did not analyse *Permanent Repercussion on Sexual Activity* because 87% (n = 208) of G1 victims did not refer to this type of damage (OR = 2.7, 95% CI 1.7–4.3).

### 2.3. Data Analysis

All analyses were performed using SPSS for Windows Version 27.0 (IBM Corp., Armonk, NY, USA). Descriptive statistics were used to describe the study population in total and stratified by age. The chi-square test was used to assess the dependence between the frequency variables. Logistic regression was used to estimate the odds ratio (OR), considering the 95% confidence interval (95% CI) for all measures of effect analysed. The OR was statistically significant if the confidence interval did not cross the value 1. In all analyses, the level of statistical significance was set at a *p*-value of <0.05.

## 3. Results

The timespan between RTA and the final PIA was, on average, 307.1 ± 236.2 days (Min = 15; Max = 1888; OR = 0.998).

Most of the victims presented a pathologic history prior to the RTA (n = 352; 73.6%), with 91.6% in G1 and 55.6% in G2, with a significant difference (OR = 8.7, 95% CI = 5.2–14.7). Considering trauma history, only 35.8% (n = 171) of the victims presented it, with 36.8% in G1 and 34.7% in G2, without differences (OR = 1.1, 95% CI = 0.8–1.6).

### 3.1. Temporary Outcomes

The temporary outcomes are described in Table 2.

The mean *Quantum Doloris* degree was similar in the two groups, with most victims assigned to grade 3 or 4. However, in G1, the higher grades were more frequently assigned.

### 3.2. Permanent Outcomes

Using the *three-dimensional* evaluation, the description of the permanent outcome is presented in Table 3.

Concerning the *three-dimensional methodology*, permanent severe damage quantification is referred to in Table 4. All three levels assessed (*body*, *functions*, and *situations*) were much more severe in G1: 1.3, 1.7, and 1.3 more times, respectively. Dividing G1 into two groups (<75 and ≥75 years), the older group presented more *body* sequelae (OR = 1.3, 95% CI = 1.008–1.7), more *functional* outcomes (OR = 1.4, 95% CI = 1.05–1.9), and more *situational* outcomes (OR = 1.7, 95% CI = 1.3–2.2). The *damage coefficient* also showed an increase of 60% in G1, and the older group (≥75 years) also presented a higher coefficient (OR = 1.6, 95% CI = 1.2–2.1).

There was a correlation between pathologic history and the *damage coefficient* (*p* = 0.008), using the groups as a control variable.

The evaluation of medico-legal permanent damage parameters considered for this study is described in Table 5.

Regarding the totality of cases, PFD was assigned in 82.8%, with a mean of 9.6 ± 13.5 points (Min = 0; Max = 83). No correlation was found between pathologic history and PFD (*p* = 0.06), using the groups as control variables. Additionally, taking G1+G2, *Future Damage* was assigned in 20 cases, 5% (n = 12) in G1 and 3.3% (n = 8) in G2; these cases were related to intra-articular fractures or joint instability of the wrist (n = 2), hip (n = 6), knee (n = 12), and ankle (n = 1); in one case, the victim presented two different anatomical regions affected.

## 4. Discussion

The number of senior adults becoming road users and victims of RTAs is progressively increasing. This is due to an ageing population associated with seniors currently being healthier, more active, and more autonomous. However, a significant number of elderly persons present prior health conditions (physiological and/or pathological) that impair posttrauma recovery, making outcomes more severe. This circumstance constitutes a public health, as well as a medico-legal, concern [15,16,36], deserving further research.

Considering the proposals of the first multidisciplinary *Consensus Conference on Medico-Legal Assessment of Personal Damage in Older People* [36], we analysed the differences in injury outcomes between older and younger people and their relevance to PIA in the context of an insurance company. To perform this discussion, we will try to answer some questions posed in the paper resulting from the referred *Conference* [36].

This is a preliminary study of the subject.

### 4.1. Evidence That Posttraumatic Injury Outcomes Differ between Older and Younger Victims

According to Ingravallo et al., there is some evidence of the more unfavourable outcomes of traumatic injury being usually associated with ageing; this evidence considers the published systematic reviews on the subject (two of average quality, one of low quality, and two of very low quality), two non-systematic reviews (of very low quality), and experts’ opinions [36]. With the present study, we have generated evidence about posttraumatic outcomes in senior people through a comparison between two medico-legal samples. Next, we discuss this aspect in more depth, considering temporary and permanent outcomes.

#### 4.1.1. Evidence Regarding Temporary Outcomes

As expected, no relevant differences were found between G1 and G2 in terms of temporary damages, given that this study used propensity–ISS score-matched samples to ensure a similar degree of acute injury severity (Table 1 and Figure 2). That is, the cases started from the same degree of injury severity to assess whether they evolved differently according to age.

However, despite an absence of a significant OR, we noticed a tendency towards more days of *Total Temporary Functional Deficit* in the older population, in agreement with other authors [18,28] and as we found in our previous study without matching ISS samples (*p* = 0.001) [25]. Nevertheless, we should note that seniors are more vulnerable to bed rest and reduced food intake, which should be avoided due to the effects of immobilisation (loss of strength and muscle mass, decrease in aerobic capacity, and functional decline); such effects already appear with just ten days of bed rest [43,44,45].

A significant difference was found for *Partial Temporary Functional Deficit*, with a higher OR in G2. This result was also found in a previous study (*p* = 0.005) [25] and may be related to the more demanding physical rehabilitation period in younger people, given the greater need for their activity (Table 2).

Although the literature reports that the elderly have greater resilience to pain than adults [5,16], in our first study [25], a higher *Quantum Doloris* average was found in the elder group (*p* < 0.001), but in the current study, no significant differences were observed (Table 2). This last result was expected, considering that the concept of *Quantum Doloris* includes the severity of acute injuries, which was forcibly similar in both groups [42].

#### 4.1.2. Evidence Regarding Permanent Outcomes

Despite the similar injuries severity in the acute phase (Table 1 and Figure 2), the results showed a more significant negative evolution of the permanent outcomes in older adults (Table 3). This fact is widely reported in the literature but with very little evidence [1,14,19,20,31,32,45]. Our analysis derives from the results of the application of a tool used in Portugal, the *Inventory for Handicap Assessment* [35] (*three-dimensional methodology of PIA*), as well as of the Portuguese permanent damage parameters used in civil law.

Regarding the *three-dimensional* damage assessment, our results showed the following:

(a) *Body* sequelae: they were more frequent and more severe in G1 (Table 3 and Table 4), increasing with age, especially from the age of 74. The most frequent sequels were orthopaedic, as occurs in the general cases of RTAs [25], but in G1, they were 1.7 times more frequent than in G2 (Table 3). Regarding neurological and psychiatric sequelae, the results did not show significant differences between groups, which may be associated with the small number of these cases in each group.
(b) *Functional* outcomes: G1 presented significantly more deficits in some of the analysed functions than G2 (Table 3) and greater severity outcomes (Table 4). *Functional* outcome severity was even higher than the respective *body* sequelae severity: 1.7 and 1.3 times more, respectively (Table 4). This leads us to consider that in this age group, any physical sequel, even a minor one, can have a high functional impact. Regarding chronic pain, we did not find differences between groups, perhaps because the number of cases was small (Table 3).
(c) *Situational* outcomes: G1 showed significantly more difficulties in the acts of daily life than G2 (Table 3). The severity of the outcomes was 1.3 times higher in G1 (Table 4). Following these real-life situations, G1 also presented a high OR for *permanent needs*, especially for third-party dependence (loss of autonomy);
(d) *Damage Coefficient*: it represents the average of *body*, *functional* and *situational* outcomes, shown to be 1.6 times more likely to evolve negatively in G1 than in G2, particularly for victims older than 74 years.

These results are well explained from a physiopathological point of view in the scientific literature. Physical health decline observed in older adults increases the probability of poor outcomes among these persons when they are victims of trauma. Even minor trauma can present more severe implications for a senior person when compared with a younger person who suffers the same injury [5,14,15,20,23,32,33,34,36].

Considering orthopaedic trauma, more frequent in RTAs [25], the bones of older people are less resistant, which is associated with a decrease in muscle mass (linked with age evolution), causing less resilience to traumatic mechanical forces. These effects act synergistically with some pathologies, such as osteopenia, osteoporosis, and sarcopenia, with the incidence of fractures even higher in persons with these health conditions. These comorbidities also make the recovery and return to the previous state more difficult as they reduce the physiological functional reserve [1,14,19,20,31,32,45].

Neurocerebral and psychiatric posttraumatic consequences are complex subjects, particularly in older persons. Associated with ageing, there is a gradual loss of cognitive abilities, explained by several factors, including changes in brain plasticity [2]. Traumatic events, even mild ones, can trigger or accelerate senile evolution due to the decrease in neurocognitive capacity; thus, cognitive changes can appear, such as distorted consciousness, confusion, altered attention, thought, perception, and memory [20,32,46,47,48,49]. A return to the previous mental state often occurs within a few days of the causal event. Nevertheless, it can also initiate neuropsychological dysfunction, which can progress to pseudodementia or dementia and can unmask previously unknown mental health conditions, leading to a significant reduction in health and functional status, with loss of autonomy and independence, and, consequently, a decrease in quality of life after trauma [20,46,50]. Studies have also shown a relationship between physical impairment and adverse psychiatric outcomes in people with a prior history of mental illness [50], with an increased disability expected in these cases [32,47,48,49]. In addition, the loss of skills and functionality, affecting daily activities, can complicate and exacerbate the already existing mental issues [46], working like a snowball mechanism.

The literature suggests that older adults react better to chronic pain than younger adults, with middle-aged adults showing higher rates of catastrophic chronic pain [16] and a higher rate of depression associated with chronic pain than older adults [27]. These findings indicate that elderly individuals tend to be more tolerant of pain than younger individuals. Another study states that older adults react to actual pain intensity, while younger adults associate pain with emotional response [5]. However, persistent pain after trauma is frequent in older adults and is associated with functional decline and activity limitations, including in daily life tasks [33].

Functional capacity is an essential indicator of geriatric health because this deficit results in the loss of independence/autonomy and is a predictor of mortality [51]. As said before, even minor or mild injuries in the elderly can lead to loss of skills and activities/participation regarding daily living tasks in previously independent and autonomous older adults [52]. Two years after the RTA, injuries continue to negatively impact the daily life of seniors, being associated with tiredness and increased physical limitations, decreased skills to perform activities of daily living, and consequences on social life [5,23,53,54]. However, there are some positive predictors for returning to independent living after trauma, such as improvement in cognitive function, mobility, and nutritional status [51]. However, some reasons may justify why seniors limit their activities after trauma, such as: (a) the assumption that the loss of autonomy after trauma, in this age group, is a natural outcome; and (b) the fear of reinjury, mainly by the risk of falling, sometimes increased after trauma. This is a cause of reduced independence and autonomy in the activities of daily living [55], with higher rates of third-party dependence.

### 4.2. The Medico-Legal Relevance of More Severe Posttraumatic Outcomes in Older Adults than in Other Persons

The results of the present study led us to believe that the PIA methodology traditionally used may not be adapted to the correct evaluation of these cases in older people.

Medico-legal PIA rules vary between countries, according to each legislation and forensic organisation. Some do not have concrete rules for PIA, but others do, as happens in Portugal [40,41,42]. Nevertheless, most countries consider temporary and permanent damages with the respective damage parameters [56,57,58,59,60]. In general, the most valued parameters are those related to PFD and *Permanent Professional Repercussion*.

In the current study, PFD did not show significant differences between G1 and G2, despite increasing with age. This result was not expected, taking into account everything that has been said previously about the physiopathological aspects of trauma in the ageing process. This was also not expected, considering the results obtained with the *three-dimensional methodology*. It can, therefore, be considered that there is no total correlation between the evaluation obtained through the *three-dimensional methodology* and the use of the Portuguese table of permanent disabilities (Decree-Law 352/2007, 23rd October, annexe 2) to assess the PFD (compare the results of Table 4 and Table 5).

We consider that one reason for these results may rely on the use of a table of permanent disabilities, which tends to value the *body* sequelae more than the *functional* and *situational* outcomes. A table of disabilities is a standardised tool that does not allow for personalised and comprehensive evaluations. On the contrary, functions and, particularly, situation descriptions can translate the reality of the aftermath of trauma regarding a person’s reality in his or her different contexts of life and participation. Therefore, the PFD also needs to reflect these aspects, and experts should be attentive to correctly quantify this damage parameter, as well as others, particularly in seniors, avoiding limiting the PIA to the use of a table of permanent disabilities [36].

Another reason we consider is the previous health state of the victim; the group of older people necessarily presents more physical and mental weaknesses related to the physiological process of ageing. Simultaneously, this group presented high rates of pathologic and trauma history (92% and 37%, respectively, in our study). Thus, it is possible to admit that it is sometimes difficult to distinguish, in the analysis of the previous state, which aspects are due to ageing, a prior disease, or trauma (e.g., joint arthrosis). This fact can lead the experts, when using a table of disabilities, to determine the PFD with a greater focus on *body* sequelae based on what was expected, given the injury that gave rise to it. On the other hand, if the expert considers that the previous state has worsened or was aggravated by the sequelae of the trauma, the PFD will be less valued than if everything resulted from it. These aspects may explain why the PFD has not increased proportionally to the different levels of the *three-dimensional* methodology and the *damage coefficient*. This is a key point on this subject, which will deserve a discussion later in this paper.

Consequently, we think that a global and personalised PIA is compulsory, especially in senior cases, to correctly contextualise all aspects related to changes due to age.

*Permanent Aesthetic Damage* was the only parameter of damage assessed that was significantly different between groups, being less valued in G1 than in G2 (Table 5). This is understandable because older people do not have the same exigences as young people in this matter, but we must be careful with this aspect, respecting the perception of each person, independent of his or her age.

Considering *Permanent Repercussions of Sporting and Leisure Activities*, we have found no differences, although G1 was higher, which may be due to the contrariety/frustration related to the loss of independence and autonomy (Table 5).

Other damage parameters were not analysed for the reasons described in the Materials and Methods chapter, but increasingly, issues such as sexuality and work activity are also relevant for older people. Ignoring these aspects just because of age can be considered ageism, and experts should take this into consideration.

### 4.3. Time of the First Medico-Legal Examination in Older Adults

According to a study performed by the *Associação Portuguesa de Seguradores* [61], the minimum time interval before the injured person undergoes medico-legal evaluation may depend on the severity of each case and the scope of the expertise. The date of the first assessment of damage by the insurer’s medical expert should occur within 30 days after the accident, whenever possible. This first evaluation aims to: (a) assess the quality/suitability of the provided treatments (including rehabilitation measures); (b) forecast future damages, which allows the insurer to make a provision; (c) signal possible needs (e.g., support products, home adaptation, third-party assistance); and (d) contribute to the preparation of clinical discharge (return to home). Follow-up evaluations will follow, which must be conducted by the same expert who carried out the first evaluation, with the appropriate frequency for each case, and maintain the same objectives mentioned above. During this entire period, if necessary, rehabilitation and reintegration teams are mobilised to evaluate and prepare an intervention plan, chosen, as far as possible, by agreement between the insurer and the victim or his or her legal representative. When the clinical stabilisation of the injuries is reached and the rehabilitation process related to the acute phase is concluded, the medical-legal expert will carry out the final PIA, which aims to describe and quantify the temporary and permanent damages (including economic and noneconomic damages) as well as identifying permanent needs (e.g., support products; home, workplace, and/or vehicle adaptations; third-party support). According to our study, the final PIA requires an average of 300 days to take place (Min = 15; Max = 1888).

If there is no agreement between the parties, there will be a legal process, and in this case, expertise at the National Institute of Legal Medicine and Forensic Sciences (INMLCF) will be mandatory, which, in principle, should only take place after the medico-legal consolidation. According to our study, the consolidation time (*Total Temporary Functional Deficit* plus *Partial Temporary Functional Deficit*) is, on average, 200 days (Table 2).

### 4.4. Number of Medico-Legal Examinations in Older Adults

The number of examinations vary according to their context.

At Portuguese insurance companies, after the first evaluation, the necessary follow-up consultations should be conducted with the appropriate frequency for each case [61]. When the medico-legal consolidation of the injuries is reached, the expert may perform the final PIA [61].

At the INMLCF, there may be several evaluations, even if the medico-legal case is consolidated. This is because the expert responsible may request ancillary exams or specialised medical advice, or, in some rare cases, the injuries may not have consolidated yet [40,41,42].

### 4.5. Duration of the Medico-Legal Examination in Older Adults

This aspect varies, obviously, according to the demand and necessity of the victim. The more complex the case, the longer it may be. It also varies with the type of examination (preliminary, follow-up, or final). According to our experience, the average time is 1.5 h if we consider that there will be more than one examination during the recovery time. In severe cases, 2 h may not be enough; however, the patient may not be able to handle more than that, so a new appointment may have to be made to complete the evaluation.

### 4.6. Logistical Conditions of the Medico-Legal Examination in Older Adults

Regarding the logistical issues of the visit, in Portugal, this can occur in an inpatient setting, in the patient’s home or institution, via video call, or in the expert’s office. We believe that video call consultations can be useful to maintain a regular follow-up of these persons, avoiding the inconvenience related to their displacement, especially when large distances and mobility problems are involved; however, they can only take place in some moments of the follow-up, and in most cases, elderly victims need the support of someone to use the informatic tools. In the case of the expert’s office, it will be important to ensure a comfortable space with adequate dimensions to admit a patient on a litter or in a wheelchair as well as an observation table that allows height adjustment. A special mention must, however, be made concerning evaluating people in their real living spaces. The victim’s inhabiting environment is one of the most significant determinants of his or her situation and participation [62]. It can offer both barriers and resources that can alter the individual’s autonomy and involvement in activities that interest him or her. The association between the individual’s intrinsic capabilities and the characteristics of their life-space and their interaction are fundamental in determining his or her functional abilities and the degree of independence and autonomy. Thus, in the more complex and severe cases, these evaluations should be conducted at the victim’s home or nursing home as part of a multidimensional assessment program for older adults [3,36].

### 4.7. Accompanying Person of the Older Adult Examination

In Portugal, the family member or caregiver can attend the medico-legal visit whenever the victim wants them to or whenever the victim is unable, by his or her means, to participate in the clinical interview. In principle, they will only be able to assist and provide the necessary support to the victim, not being authorised to intervene. However, especially in the case of older people, whether due to cognitive, hearing, or other problems, we consider that the intervention of the family member or caregiver can be beneficial for the expert to provide the necessary information or complement/validate the information given by the victim.

We also consider that to perform the physical examination, the expert must ask the victim again whether he or she still wants the presence of the accompanying person. If this person remains present during the physical examination, his or her assistance in, for example, walking or undressing/dressing clothes, should only be consented to after the expert has observed the victim trying to do so by his or her own means.

According to our opinion, this presence should be registered in the medico-legal report.

### 4.8. Access to Clinical Information about the Case

The information required from the victim and/or his or her accompanying person will be related to the circumstances of the accident, his or her personal trauma experience, the resulting injuries, the subsequent treatments and eventual hospitalisations, medical consultations, ancillary exams, and rehabilitation therapies, in addition to the information about his or her pathologic/traumatic history (previous status) [40,41,42]. The clinical records will later confirm and complement all this information [20,42]. For this last purpose, in the case of private medical practice, in Portugal, the victim or his or her legal representative can provide the clinical records to the expert, authorising them to consult and transcribe what they deem appropriate. If they do not have these clinical elements, a written authorisation can be given to the insurance company or the physician to request these documents directly from the respective institutions. In this study, the experts had access to the needed information.

According to the law (Law 45/2004, 19th August, actualised by the Decree-Law 53/2021, 16th July), in the INMLCF case, an expert may directly access these documents within the public services or request them from other institutions without the need for consent by the victim or his or her legal representative.

The victim must also respond to his or her complaints in functional and situational terms through questions that should be as open as possible and not suggestive. To describe the complaints, in Portugal, medical experts follow the *three-dimensional* PIA model [35]. In the case of the first exam and follow-up exams, the medical expert must, at the end of the visit, inform the victim about his or her clinical evolution and the therapeutic and support procedures that will follow, verifying if there is motivation and agreement from the victim. In the case of the last visit, the expert must explain which route will be given in the medico-legal report at the end.

### 4.9. Access to Information about the Previous State

As said before, the previous health state is a crucial point in PIAs, mainly what older adults are concerned about. Therefore, the medico-legal expert must always collect the preinjury status.

This may be possible through information directly provided by the victim or whoever represents him or her [20]. However, the victim often does not understand the objectives of the medical-legal evaluation and may act with reserve or suspicion and might, consciously or not, act with simulation, dissimulation, or exaggeration of his or her complaints. Thus, the victim can conceal his or her previous status, not collaborate with the physical exam, or report overstated complaints and disabilities, with a discrepancy between these and the physical exam as well as the ancillary exams performed.

Therefore, the experts must have access to clinical medical records and other documents that could inform them about the previous situation of the victim. Nevertheless, in Portugal, if acting in public service (INMLCF), the experts have the legal right to access clinical documentation (Law 45/2004, 19th August, actualised by the Decree-Law 53/2021, 16th July); the same is not valid for private services (including insurance companies) due to the right of privacy and to the Personal Data Protection Law (Decree-Law 58/2019, 8th August). Thus, this aspect can be a complex problem, given that most PIAs related to RTAs take place using insurance companies’ medical services. In this way, to achieve a reliable reconstitution of the previous health state, the medical experts of the insurance companies must explain to the victims and/or to their representatives the importance of providing all the documents needed (including reports from the general practitioner physician), authorising their consultation and description in the medico-legal report. This may promote the extrajudicial resolution of the case instead of a court resolution, which further increases the costs of the process and delays the conclusion of the case. In the present study, much of the information considering the previous state was obtained through the hospital clinical reports related to the accident, where the pathologic history is usually described. Additionally, several times, the physical examination and ancillary exams allowed us to obtain important information about prior conditions.

The information needed should include, among others, the following [20,36,42]: (a) pre-existing pathologies, malformations, and previous trauma history; (b) pharmacological, psychological, or rehabilitation therapies; (c) history of regular consultations, and previous ancillary exams; (d) ageing process and related frailties; (e) former functional status, including cognitive performance (memory impairments, education status, mental disabilities, or other problems); (f) independence level and personal autonomy degree (ability to perform autonomously personal care, autonomy in activities of daily living, domestic tasks); (g) consumption habits (alcohol, tobacco, and other drugs); (h) nutritional conditions (type of alimentation, including weight); (i) lifestyle (physical activities, hobbies, sports, recreational tasks, among others); (j) family context and affective state; (k) social relations, interactions, and leisure activities; and (l) professional activities among others with economic relevance.

Nevertheless, according to our experience, all this information may not be obtainable just through medical documents, and therefore, a multidisciplinary assessment, including the participation of social workers and psychologists, in certain cases at the victim’s place, can be extremely helpful in informing the expert [61].

### 4.10. Aspects to Be Considered in Damage Assessment in Older Adults

The expert should perform a personalised and comprehensive evaluation, including *functional* and *situational* complaints and the results of the *body* examination [42]. The confrontation of the findings between them and with the mechanism of the trauma, injuries, treatments and clinical complications may orientate the expert to determine the date of consolidation and the causality link [63]. However, we consider that the expert should always have in mind that in older person cases, this should not be enough, and deep information about the previous state should be sought, as well as a multidimensional/multidisciplinary examination in loco performed, particularly in more severe or complex cases.

### 4.11. Ancillary Exams in Older Adults

These exams can be very useful not only for the current medico-legal diagnosis of lesions but also for detecting previous pathologies or sequelae that should be considered. In the cases where the medico-legal expert requests an exam, the patient or his or her legal representative needs to give the necessary informed consent.

### 4.12. Causal Link in Posttraumatic Cases of Older Adults

Our practice shows that one of the great difficulties that can be felt by medico-legal experts in the PIA of senior persons may be related to the link between trauma/injuries and the effective permanent outcomes, especially *functional* and *situational* outcomes, which often seem to be disproportional to *body* sequelae. This creates problems in the discussion and admissibility of the nexus link and the assignment of the medico-legal damage parameters, including the real permanent needs of the older person. However, it is overly noted in the literature and this study that older adults can lose their autonomy and become third-party-dependent when suffering even minor injuries [24,45,52]. Thus, when evaluating elderly persons, medico-legal experts should perform a comprehensive and personalised assessment to try to understand the whole picture, considering the real context of the person’s life and avoiding limiting the PIA to the injury or *body* sequelae evaluation [20,40,42], always keeping in mind the evidence regarding the physiopathology of trauma in elderly persons.

The expert must analyse the various assumptions involved in its evaluation [42]: (a) consistency between the type of injury or sequelae and the concrete trauma dynamics; (b) consistency between the type of trauma and type of injury or sequelae incurred; (c) consistency between the site of the trauma and the site of the injury or sequelae; (d) anatomical–clinical consistency between the trauma and injury or sequelae; (e) temporal consistency between the event, injury, and sequelae; (f) exclusion of the possibility that the injury or sequelae may have pre-existed; and (g) exclusion of the possibility that the injury or sequelae may have been caused by a mechanism other than the event.

In the case of older people, each of these assumptions is a challenge in our daily clinical practice, but the most difficult will often be relative to the previous state. Fundamentally, the discussion of the anatomical–clinical consistency between the trauma and sequelae/outcomes will mean that the expert will be able to explain why he or she accepts the nexus with some outcomes and not with others [63]. For this, we advise the experts to always consider the victim in his or her real and specific life context, as well as to be aware of the current knowledge regarding the effect of trauma on the ageing process, which can lead to unexpected and serious developments.

Thus, it is essential to consider not only the specific previous diseases and comorbidities, along with the current sequelae, but also how they interact and impact each other and with the victim’s functionality and integration into his or her environment, which is also a much better predictors of survival and other outcomes [4,36].

In a simplistic way, we could say that the expert should compare the victim’s previous state (considering all the above information) with the posttrauma status. The difference between the previous and the current status should be considered to correspond to the damage resulting from the accident. As mentioned before, even minor or mild injuries and sequelae in older people can evolve from a state of total autonomy to a status of dependence with a loss of autonomy [24,45,52]. Furthermore, one must always keep in mind that age is not a co-cause [20], although this consideration deserves a special discussion due to the fact that old age is currently included in the ICD-10 [8]. In any case, as demonstrated in this study, trauma does contribute to worse outcomes in older people, and the medical expert must know the physiopathological consequences of trauma in the ageing process and accept them in the causal nexus, totally or partially, since this evolution is clinically reasonable and explainable.

### 4.13. Consolidation Date in Older Adults

In general, consolidation in older persons should be considered when no further evolution of the injuries is expected, as it happens in other cases [40,41]. However, in more complex or severe clinical situations, physical and functional consolidation may not be enough. In these cases, the medico-legal expert should wait for the reorganisation of their livelihood to be carried out, as well as the reintegration process [42].

According to our study, the consolidation time (*Total Temporary Functional Deficit* plus *Partial Temporary Functional Deficit*) is, on average, 200 days after the RTA for G1, while it is higher for G2. Our practice shows that this date sometimes may be assigned earlier than for younger people, as the demands in terms of the outcome of rehabilitation may not be very high for the type of daily activity they will have. On the other hand, many of these people will maintain rehabilitation treatments regularly for the rest of their lives, which does not mean that the consolidation date is extended indefinitely. Furthermore, it is often important for the victim to complete the insurance process, which is regularly associated with the consolidation date. Another relevant decision, in some cases, is about a new living place for that person. In any case, we consider that consolidation should not be assigned before all these logistical aspects are well defined and implemented, an aspect that may substantially extend the consolidation date in the more severe cases.

### 4.14. Assessment Tools in Older Adults

In Portugal, we use the *three-dimensional methodology* to describe the sequelae [35,40,41,42]. However, more important than a tool is that the medico-legal expert rigorously and systematically describes all of the complaints in functional terms and in the patient’s real-life situation, which must be compared with the results of the physical examination and other eventual ancillary exams, to assess the feasibility of these complaints.

To quantify the damage, we use a scale of 1–7 degrees of increased severity for noneconomic damages and a table of permanent disabilities in civil law to determine PFD (Decree-Law 352/2007, 23rd October, annexe 2).

The results of the use of these instruments may not always be well aligned, especially in the case of older people, as verified in this study. Therefore, we think that the medico-legal expert must use them in a complementary way, with his or her clinical and medico-legal judgement always prevailing, taking into account the concrete reality of the case and the result of other forms of evaluation that he or she may resort to.

### 4.15. Multidisciplinary and Multidimensional Assessment in Older Adults

A multidimensional assessment of older persons should be undertaken, especially in severe cases and where accessibility and mobility are concerned [62]. However, we believe that other aspects must also be considered, such as social isolation, poverty, and difficulties in familial dynamics. In this sense, the multidimensional assessment requires a multidisciplinary approach, that is, not only by the medico-legal expert but also with the contribution of other professionals, whose expertise may vary according to the specific case (e.g., social worker, psychologist, rehabilitation nurse, ergo-therapist, engineer, architect). This in loco evaluation may allow, in addition to the physical and functional assessment, the real analysis of the situational state, such as in activities of daily living, evaluating the ability to perform basic activities (e.g., eating, mobility, use of the toilet, dressing, and bathing), instrumental activities (e.g., using the telephone, shopping, preparing food, taking care of the house, washing clothes, using transportation, taking responsibility for his or her medication, and taking care of his or her economic affairs), and advanced activities (e.g., cultural, recreational, and professional activities) [20,35,36,42].

Our experience shows that these collaborations may also allow a more comprehensive evaluation and help the expert to prepare a medico-legal report that, in addition to the assessment of the various damage parameters, also considers the real temporary and permanent needs of the victim in his or her concrete life contexts. Moreover, the identification in loco of the various difficulties and limitations resulting from the RTA for the victim will better allow the expert to substantiate the causal link between the trauma suffered and the resulting outcomes. This reasoning, when decontextualised from the reality of each individual, may not be sufficiently enlightening and may lead to the underestimation of the true damage suffered by seniors.

In more complex cases, the evaluation of mental status must be performed by a psychiatrist and/or a psychologist. We consider that this, perhaps, the most difficult part of the older person assessment and of causal link discussion because even without brain injuries, intellectual functions (such as attention, memory, spatial perception, orientation, judgement capacity, ability to expose and solve problems, language and psycho-affective framework, maintenance of interests, sleep quality, mood, and sense of well-being) may decline or be aggravated by injuries, trauma experience, hospitalisation, removal from home and family, and the interruption of life routines, among others. This functional mental damage will affect daily life, social and leisure, and relational activities [20,42,46,47,48].

Many of the aspects covered in our study are already based on information resulting from hospital, home, and video consultations prior to the last consultation, which, the vast majority of the time, is at the doctor’s office.

## 5. Limitations of this Study and Further Studies

The limitations of this study were: (a) the use of a convenience sample with a relatively small size, which was not possible to avoid in the current study, considering the available data; (b) the fact that the analyses of the temporary outcomes and the higher vulnerability of the older adults to the initial trauma impact were impaired by the study’s design (match of the ISS); (c) the delimitation of the study to RTAs and to the Portuguese context in civil law; (d) the absence of the analysis concerning the number of medico-legal examinations that the victims underwent and the respective duration, as well as the concrete difficulties in access information about the previous state of the victim; and (e) the absence of a validated tool to assess older adults in the *three-dimensional* perspective, although we used a validated inventory for RTA outcomes assessment in a medico-legal context [35].

Further research should consider: (a) a real-world, retrospective, observational, and cross-sectional study on this subject using a federated data analysis methodology, which may constitute an essential contribution to a better understanding of these complex cases; (b) a study including and comparing other countries to understand if there are differences when considering other realities; (c) a study focusing on a multidimensional and multidisciplinary approach in these cases; (d) a study considering another kind of trauma (e.g., occupational accidents); and (e) the validation of the *Inventory for Handicap Assessment* for individuals over 64 years old.

## 6. Conclusions

The present study used a sample of RTA victims aged 65 years or more (G1), which was compared with another sample of younger victims (G2), assuring a similar degree of acute injury severity in both (using propensity–ISS score-matched samples). We verified the following:

(a) In G1, 92% had a pathological history (8.7 times more than G2) and 37% had a trauma history;
(b) No relevant differences were found between G1 and G2 in terms of temporary damages, as expected, given that both age groups had a similar degree of acute injury severity; an exception was found for G2, which had a higher *Partial Temporary Functional Deficit* than G1 (OR = 0.99);
(c) Regarding the *three-dimensional methodology* evaluation of permanent outcomes, G1 presented more sequelae than G2: (a) *body* sequelae—orthopaedic (OR = 1.7); (b) *functional* outcomes—carriage, displacement, and transfers (OR = 1.6), manipulation and grip (OR = 1.8), and sphincter control (OR = 3.8); and (c) *situational* outcomes—acts of daily life (OR = 2.9); G2 had more nonexistent *body* sequelae (OR = 0.5) and *functional* outcomes (OR = 0.4);
(d) All three levels of damage severity (*body*, *functions*, and *situations*) and the *damage coefficient* were much more severe in G1 (1.3, 1.7, 1.3, and 1.6 more times, respectively);
(e) G1 presented more *Permanent Needs* than G2 regarding third-party assistance (OR = 3.5), medication (OR = 2), and technical aids (OR = 2.4); G2 evolved more without any *Permanent Needs* (OR = 0.4);
(f) There was no difference in the *Permanent Functional Deficit* between the two age groups, although G2 was assigned more grade 0s (OR = 0.5) and G1 more grade 2s (OR = 1.8);
(g) G2 was assigned more *Permanent Aesthetic Damage* than G1 (OR = 0.9).

This study offers evidence that the consequences for older victims of RTAs are more severe than for younger counterparts. Based on these results and the literature, we offer some contributions to set the grounds for posttraumatic senior PIA guidelines.

## Figures and Tables

**Table 1:** Matched sample characterisation regarding ISS.

	ISS	Total (n = 478)	G1 (n = 239)	G2 (n = 239)	Standardised Difference
$\overset{-}{X}$		11.6 ± 10.1	11.71 ± 10.4	11.51 ± 10	0.01
n (%)	1–8 (mild/moderate)	195 (40.8)	96 (40.2)	99 (41.4)	0.01
	9–15 (serious)	139 (29.1)	71 (29.7)	68 (28.5)	-0.01
	16–24 (severe)	77 (16.1)	39 (16.3)	38 (15.9)	-0.01
	≥25 (critical)	67 (14)	33 (13.8)	34 (14.2)	0.01

**Table 2:** Temporary outcomes (medico-legal damage parameters).

			G1 (n = 239)	G2 (n = 239)	OR	95% CI (Min–Max)
*Temporary Functional* *Deficit* ($\overset{-}{X}$ days)	*Total*		31.3 ± 77	29 ± 87.2	1	0.99–1.03
	*Partial*		165.3 ± 148.8	222.6 ± 215.2	0.99	0.97–0.99 *
*Quantum Doloris* (Grade 1–7)	$\overset{-}{X}$		3.9 ± 1	3.9 ± 0.9	1.04	0.85–1.3
	n (%)	1–3	72 (30.1)	86 (36)	0.8	0.5–1.1
		4–7	167 (69.9)	153 (64)	1.3	0.9–1.9

* Significant differences.

**Table 3:** Permanent outcomes (*three-dimensional methodology*).

Permanent Outcomes		G1 n (%) (n = 239)	G2 n (%) (n = 239)	OR	95% CI (Min–Max)
*Body*	Orthopaedic	191 (79.9)	167 (69.9)	1.7	1.1–2.6 *
	Neuro-psychiatric	40 (16.7)	38 (15.9)	1.1	0.7–1.7
	Others	48 (20.1)	64 (26.8)	0.7	0.4–1.1
	Nonexistent	29 (12.1)	49 (20.5)	0.5	0.3–0.9*
*Functions*	Carriage, displacement, and transfers	167 (69.9)	140 (58.6)	1.6	1.1–2.4 *
	Manipulation and grip	95 (39.7)	62 (25.9)	1.9	1.3–2.8 *
	Cognition, affectivity, and communication	61 (25.5)	52 (21.8)	1.2	0.8–1.8
	Chronic pain	42 (17.6)	29 (12.1)	1.5	0.9–2.6
	Sphincter control	18 (7.5)	5 (2.1)	3.8	1.4–10.4 *
	Senses	11 (4.6)	9 (3.8)	1.2	0.5–3
	Nonexistent	27 (11.3)	55 (23)	0.4	0.3–0.7 *
*Situations*	Acts of daily life	188 (78.7)	133 (55.6)	2.9	2–4.4 *
	Affective, social life, and leisure activities	124 (48.1)	114 (47.7)	1.2	0.8–1.7
	Nonexistent	40 (16.7)	51 (21.3)	0.7	0.5–1.2
*Permanent needs*	Third-party assistance (partial or total)	55 (23)	19 (7.9)	3.5	2–6 *
	Regular medical treatments	30 (12.6)	22 (9.2)	1.4	0.8–2.5
	Regular medical appointment	18 (7.5)	21 (8.8)	0.8	0.4–1.6
	Medication	25 (10.5)	13 (5.4)	2	1.01–4.1 *
	Orthoses	20 (8.4)	11 (4.6)	1.9	0.9–4
	Technical aids	18 (7.5)	8 (3.3)	2.4	1.01–5.5 *
	Prothesis	4 (1.7)	9 (3.8)	0.4	0.1–1.4
	Consumables	7 (2.9)	4 (1.7)	1.8	0.5–6.1
	Ancillary exams	4 (1.7)	2 (0.8)	2	0.4–11.1
	Nonexistent	153 (64)	193 (80.8)	0.4	0.3–0.6 *

* Significant differences. Note: *Body*, *functional*, and *situational* outcomes, as well as *permanent needs* categories, are not mutually exclusive.

**Table 4:** The severity of permanent outcomes (*three-dimensional methodology*).

Severity (0–4)	$G1\;\overset{-}{X}$	$G2\;\overset{-}{X}$	Standardised Difference	OR	95% CI (Min–Max)
*Body sequels*	1.6 ± 1.0	1.3 ± 1.0	0.27	1.3	1.1–1.6 *
*Functional outcomes*	0.9 ± 0.9	0.5 ± 0.8	0.43	1.7	1.3–2.1 *
*Situational outcomes*	0.9 ± 1.1	0.6 ± 0.9	0.28	1.3	1.1–1.6 *
*Damage coefficient*	1.5±1.0	1.1±0.8	0.44	1.6	1.3–2.0 *

* Significant differences.

**Table 5:** Permanent medico-legal damage parameters assessed.

			G1 (n = 239)	G2 (n = 239)	OR	95% CI (Min–Max)
*Permanent Functional Deficit*	$\overset{-}{X}$	(0–100 points)	10.4 ± 13.6	8.8 ± 13.4	1.009	0.995–1.023
	n (%)	0	31 (13)	51 (21.3)	0.5	0.3–0.9 *
		1–9	117 (49)	124 (51.9)	0.9	0.6–1.3
		10–19	60 (25.1)	37 (15.5)	1.8	1.2–2.9 *
		20–39	21 (8.8)	16 (6.7)	1.3	0.7–2.6
		≥40	10 (4.1)	11 (4.6)	0.9	0.4–2.2
*Permanent Aesthetic Damage*	$\overset{-}{X}$	(Degree 1–7)	1.9 ± 1	2.1 ± 1	0.9	0.7–0.99 *
	n (%)	1	43 (18)	44 (18.4)	0.97	0.6–1.5
		2	44 (18.4)	45 (18.8)	0.97	0.6–1.5
		3	21 (8.8)	19 (8)	1.1	0.6–2.1
		4–7	5 (2.1)	20 (8.4)	0.2	0.09–0.6 *
		Nonexistent	126 (52.7)	111 (46.4)	1.3	0.9–1.8
*Permanent Rep. Sport. and Leisure Activities*	$\overset{-}{X}$	(Degree 1–7)	3.06 ± 1.7	3.13 ± 1.7	0.9	0.8–1.1
	n (%)	1	5 (2.1)	7 (2.9)	0.7	0.2–2.3
		2	17 (7.1)	7 (2.9)	2.5	1.03–6.2 *
		3	8 (3.3)	5 (2.1)	1.6	0.5–5
		4–7	13 (5.5)	13 (5.5)	1	0.5–2.2
		Nonexistent	196 (82)	207 (86.6)	0.7	0.4–1.2

* Significant differences.

## Data Availability

The results from the dataset are presented in the paper. The full dataset is available from the first author upon request.
